# Evaluation of Malnutrition and Its Association With Biochemical Parameters in Patients With End Stage Renal Disease Undergoing Hemodialysis Using Subjective Global Assessment

**DOI:** 10.5812/numonthly.16385

**Published:** 2014-05-15

**Authors:** Fatemeh Espahbodi, Talayeh Khoddad, Leila Esmaeili

**Affiliations:** 1 Imam Khomeini Hospital, Mazandaran University of Medical Sciences, Sari, IR Iran

**Keywords:** Renal Dialysis, Malnutrition, Kidney Failure, Chronic, Biological Markers

## Abstract

**Background:**

Malnutrition is a common problem in patients with end stage renal disease (ESRD) undergoing hemodialysis that increases morbidity and mortality rate in them. Subjective global assessment (SGA) is a tool used by health care providers to assess nutritional status in these patients. In addition, biochemical parameters are used to assess the nutritional status in all people.

**Objectives:**

In this study, we evaluated the nutritional status of patients with ESRD undergoing hemodialysis using SGA and assessed probable association between biochemical parameters and malnutrition in this population.

**Patients and Methods:**

Using SGA, the nutritional status of 105 patients (60 males and 45 females) of two dialysis centers in Sari, Iran, was evaluated during years 2007-2008. It is a semiquantitative scoring system that has seven variables derived from medical history and physical examination. The biochemical parameters including hemoglobin, albumin, cholesterol, BUN, and creatinine were also measured.

**Results:**

Among 105 patients, 98 (93.33%) patients consisted of 56 males and 42 females had mild to moderate malnutrition and 3 (2.86%) women had severe malnutrition. In addition, all of the patients without malnutrition were men. We found significant association between patient’s sex and the SGA score (P = 0.03) but no significant association was seen between age and duration of hemodialysis with SGA score. In addition, we did not find significant association between the measured biochemical parameters and malnutrition.

**Conclusions:**

According to high prevalence of malnutrition in our patients with ESRD undergoing hemodialysis, periodic assessment of nutritional status is necessary in them. Meanwhile we found SGA as the best tool to assess nutritional status in patients with ESRD undergoing hemodialysis, because it can recognize various degrees of malnutrition that may remain undetected by a single laboratory assessment.

## 1. Background

Malnutrition is a common problem in patients with end stage renal disease (ESRD) undergoing hemodialysis that may occur secondary to several factors such as inadequate nutritional intake, increased losses, or to an increase in protein catabolism (1-3). The results of malnutrition are various and include increased susceptibility to infection, impaired wound healing, poor rehabilitation, fatigue, malaise, and increased rates of hospitalization, morbidity and mortality (4, 5).

Subjective global assessment (SGA) is a tool used by health care providers to assess nutritional status and aids in the prediction of nutrition-associated clinical outcomes (6-8); it is inexpensive and rapidly conducted (9, 10). Moreover, it has been recommended by the National Kidney Foundation (NKF) Kidney Disease/Dialysis Outcomes and Quality Initiative (K/DOQI) for use in nutritional assessment in the adult dialysis population (11); however, it seems its semi-quantitative scale, consisting of three discrete severity levels, restricts its reliability and precision. In other words, responses to some sections of SGA depends on the patient’s ability to provide accurate data and ability of the physician to conduct detailed, probing interviews (12). Biochemical parameters like serum albumin levels are extensively used to assess the nutritional status in general population (13). Nonetheless, it seems they can be influenced by non-nutritional factors such as infection, inflammation, hydration status, peritoneal or urinary protein losses, and acidemia (14).

## 2. Objectives

Considering the importance of malnutrition in patients with ESRD undergoing hemodialysis, our main purpose was to evaluate the nutritional status of these patients in our hemodialysis centers, using the SGA score. Then, we measured patients' biochemical parameters such as albumin, hemoglobin, cholesterol, blood urea nitrogen (BUN), and creatinine as quantitative tools to assess the probable association between these biochemical parameters and malnutrition to answer this question: is it possible that we predict malnutrition based on biochemical parameters?

## 3. Patients and Methods

This cross-sectional, descriptive-analytic study was performed on all of patients with ESRD undergoing hemodialysis that were referred to the dialysis centers of Imam Khomeini and Fatemeh Zahra Hospitals of Mazandaran University of Medical Sciences, Sari, Iran, from October 2007 to October 2008. The study population composed of 105 patients (60 males and 45 females). All participants were informed of the purpose of the study and each patient signed a consent form. The patients’ information such as age, sex, the duration of hemodialysis (months), and biochemical parameters were recorded. The biochemical parameters included hemoglobin, albumin, cholesterol, BUN, and creatinine. A general practitioner under observation of a nephrologist interviewed the patients at their bedside to complete SGA.

SGA is a semiquantitative scoring system and its score are calculated based on the history and physical examination as described by Destky et al. (7) (Appendix). The history focuses on seven variables, namely weight change in preceding six months and two weeks, change in dietary intake, presence of gastrointestinal symptoms, change in functional capacity, subcutaneous loss of fat, muscle wasting, and edema. Loss of subcutaneous fat was assessed over triceps, biceps, and the fat pads below the eyes and muscle wasting was assessed on examination of temples, clavicle, and shoulder. A seven-point scoring system was applied to the above seven variables. The patients were allocated into three groups according to the points scored as follows: well-nourished or healthy (score 1-14), mild to moderately malnourished (score 15-35), and severely malnourished (score 36-49).

### 3.1. Statistical Analysis

Data were analyzed by SPSS version 16.0 for windows (SPSS Inc., Chicago, IL, USA). Parametric data were presented as mean ± standard deviation and nonparametric data were presented descriptively. Student t test, sum of squares, and Fischer’s exact test were used for data analysis. P values < 0.05 were considered statistically significant.

## 4. Results

Among 105 patients, 101 (96.19%) had various degrees of malnutrition. The level of nutritional status based on SGA score and sex distribution of the patients is shown in Table 1. We found significant association between patients’ sex and the SGA score (P = 0.03).

The patients’ age ranged from 21 to 86 years with a mean of 56.8 years and a substantial proportion (43.80%) of the patients were older than 60 years old. However, we did not find any significant association between SGA score and patients’ age (P = 0.153).

With respect to the hemodialysis duration, hemodialysis for less than 12 months were most frequent (30.04%), but we found no significant association between SGA score and duration of hemodialysis (P = 0.57) (Table 2). There was no significant association between the measured biochemical parameters such as hemoglobin, albumin, cholesterol, BUN, or creatinine and malnutrition (Table 3).

**Table 1.** Frequency of Patients Based on Sex for Three Levels of Subjective Global Assessment score ^a,b^

	7-14 (Healthy)	15-35 (Mild to Moderate Malnourished)	36-49 (Severe Malnourished)
**Male, n = 60**	4 (3.81)	56 (53.33)	0 (0)
**Female, n = 45**	0 (0)	42 (40)	3 (2.86)
**Total, n = 105**	4 (3.81)	98 (93.33)	3 (2.86)

^a^ Data are presented as No. (%).

^b^ P = 0.03.

**Table 2.** Frequency of Patients Based on Hemodialysis Duration for Three Levels of Subjective Global Assessment ^a,b^

Dialysis Duration, mo	7-14 (Healthy)	15-35 (Mild to Moderate Malnourished)	36-49 (Severe Malnourished)
**1-12**	2 (1.90)	37 (35.23)	2 (1.90)
**12-24**	2 (1.90)	14 (13.33)	0 (0)
**24-36**	0 (0)	13 (12.38)	0 (0)
**36-48**	0 (0)	10 (9.52)	0 (0)
**> 48**	0 (0)	15 (14.28)	1 (0.95)

^a^ Data are presented as No. (%).

^b^ P = 0.57.

**Table 3.** Frequency of Patients Based on Nutritional Status According to Subjective Global Assessment and Biochemical Parameters ^a^

	Healthy	Malnourished	P value
**Hemoglobin**			P > 0.05
< 11	2 (1.90)	77 (73.34)	
≥ 11	2 (1.90)	24 (22.86)	
**Albumin**			P > 0.05
< 4	1 (0.95)	80 (76.19)	
≥ 4	3 (2.86)	21 (20)	
**Blood urea nitrogen (BUN)**			P > 0.05
< 60	2 (1.90)	17 (16.2)	
≥ 60	2 (1.90)	84 (80)	
**Creatinine**			P > 0.05
< 10	4 (3.81)	80 (76.19)	
≥ 10	0 (0)	21 (20)	
**Cholesterol**			P > 0.05
< 150	3 (2.86)	63 (60)	
≥ 150	1 (0.95)	38 (36.19)	

^a^ Data are presented as No. (%).

## 5. Discussion

The main aim of this study was to evaluate of nutritional status of patients with ESRD undergoing hemodialysis in our hemodialysis centers and possibility of malnutrition prediction based on biochemical parameters in them. In our study, among 105 patients, 96.19% suffered from various degrees of malnutrition. This is nearly similar to findings of Janardhan et al. study in India. Using SGA, the malnutrition rate was 91% among 66 patients undergoing hemodialysis in their study (15). In another study by Tayyem et al. in Jordan, the malnutrition rate was 61.8 % among 178 patients undergoing hemodialysis (16). A study by Afshar et al. detected malnutrition in 40.7% of 54 patients undergoing hemodialysis in capital city of Iran (17). These differences in prevalence may be because of environmental diversity and different diet regiments in various regions.

In our study, men had no malnutrition while all women had some degrees of malnutrition. In addition, it is noteworthy that all patients with severe malnutrition were women. Our findings indicated a significant association between patients’ sex and the SGA (P = 0.03); similarly, a study in Iran by Farrokhi et al. showed that malnutrition was significantly more frequent in women (18). It appears that we should pay more attention to the nutritional status of female patients with ESRD undergoing hemodialysis.

We found no significant association between duration of hemodialysis and SGA score. It may be attributed to the greater mortality rate in patients with longer duration of hemodialysis, which reduced their proportion in our study population, or the fact that longer duration of hemodialysis improves the patients’ knowledge of their nutritional needs. In addition, our study showed no significant association between SGA score and patients’ age (P = 0.153).

We did not find any significant association between hemoglobin level and malnutrition. This is similar to findings of a study by Gurreebun et al. on 141 patients undergoing hemodialysis in England. It is probably is due to the other factors that affect hemoglobin level in patients under hemodialysis such as reduced erythropoietin production in unhealthy kidneys, severe hyperparathyroidism, acute and chronic inflammatory conditions, aluminum toxicity, reduced lifespan of red blood cells, and concomitant conditions like hemoglobinopathies, hemolysis, and limited access of patients to the recombinant erythropoietin or erythropoiesis-stimulating agents (ESAs) (19).

The association between serum albumin level and malnutrition was not significant in our study, which is similar to findings of Tapiawala et al. study on 81 patients in India (20); however, it is contrary to an American study on 52 patients, by Eustace et al. (21). Our study findings were probably due to the effect of other factors such as proteinuria, which is frequent among renal failure patients, on serum albumin level. In addition, dialysis can decrease plasma albumin level. Moreover, since albumin is an acute phase reaction protein and most patients under hemodialysis have various degrees of vascular inflammation, serum albumin level may be altered. Acidemia and hydration are other factors that affect serum albumin level. Thus, when we consider serum albumin level as a nutritional marker, it is necessary to evaluate the patient's clinical status such as concomitant conditions, quality of dialysis, acid-base status, and degree of proteinuria (22).

Our findings indicated no significant association between malnutrition and BUN level that was inconsistent with findings of a Turkish study by Afsar et al. on 137 patients (23). This may be because the urea modeling depends on many assumptions, such as constant protein intake (24). In addition, we found no significant association between malnutrition and serum creatinine level that contradicted the findings of the study aforementioned (23). Moreover, we found no significant association between serum cholesterol level and malnutrition; it is probably due to this matter that cholesterol level as an indicator of energy-protein status is insensitive, unspecific, and is affected by other factors such as inflammation (25).

Therefore, in our study, lack of significant association between biochemical parameters (such as albumin, hemoglobin, cholesterol, BUN, and creatinine) and malnutrition revealed that these parameters could not provide accurate information about nutritional status of these patients. Moreover, SGA semiquantitative scale can still be the best tool to assess nutritional status in patients with ESRD undergoing hemodialysis, because it can recognize various degrees of malnutrition that may remain undetected by a single laboratory assessment.

According to our findings that showed high prevalence of malnutrition in patients with ESRD undergoing hemodialysis and the importance of malnutrition as a prognostic factor in these patients, we recommend below items to prevent, diagnose, and treat malnutrition in patients with ESRD undergoing hemodialysis:

- Organizing continuous classes in order to educate patients with chronic renal failure who need hemodialysis about correct nutrition; in addition, periodic nutrition consultations with a dietician and provision of a detailed diet plan for each patient is very helpful.
- Conducting similar studies periodically to follow up the nutritional status of patients and success rate of interventions.
- Conducting studies to assess other methods and markers, which evaluate nutrition more accurately.

## Appendix

Standard questionnaire of subjective global assessment (SGA)

I. weight change over past two weeks and last six months

- Weight gain, no change, mild weight loss (> 0.5 kg but < 1 kg) 1-2

- Moderate weight loss (> 1 kg but < 5%) 3-5

- Severe weight loss (> 5%) 6-7

II. Change in dietary intake

- No change or slight change for a short duration 1-2

- Intake borderline and increasing 3-5

- Intake borderline or poor and decreasing 6-7

III. Presence of Gastrointestinal symptoms

- Few intermittent or no symptoms 1-2

- Some symptoms for > 2 weeks or severe symptoms that are improving 3-5

- Symptoms daily or frequently > 2 weeks 6-7

IV. Functional state

- No impairment in strength/ stamina or mild to moderate loss and now improving 1-2

- Mild to moderate loss of strength/ stamina in daily activity or severe loss but now improving 3-5

-Severe loss of strength/stamina or bed ridden 6-7

V. Subcutaneous loss of fat

- Little or no loss 1-2

- Mild-moderate in all areas 3-5

- Severe loss in some or most areas 6-7

VI. Muscle wasting

- Little or no loss 1-2

- Mild-moderate in all areas 3-5

-Severe loss in some or most areas 6-7

VII. Edema

- Little or no edema 1-2

- Mild-moderate edema 3-5

- Severe edema 6-7

Minimum score = 7, Maximum score = 49. 1-14, well nourished; 15-35, mild to moderate malnourishment and 36-49, severe malnourishment.
